# Synergetic effect of lauric acid and tea tree oil-loaded solid lipid nanoparticles and photobiomodulation in diabetic wound healing

**DOI:** 10.1007/s10103-025-04673-8

**Published:** 2025-10-11

**Authors:** Fezile Motsoene, Heidi Abrahamse, Sathish Sundar Dhilip Kumar

**Affiliations:** Laser Research Centre, University of Johannesburg, Johannesburg, South Africa

**Keywords:** Wound closure, Morphology, Laser radiation, Photobiomodulation, Diabetic fibroblastic cells

## Abstract

The management of diabetic wounds requires more than traditional care due to the challenges faced by diabetic patients, including poor circulation, neuropathy, impaired immune response, chronic inflammation, elevated blood glucose levels, and a higher risk of infection. While conventional methods often have limitations, recent studies have shown that solid lipid nanoparticles (SLNs) in combination with Photobiomodulation (PBM) are effective in promoting wound healing. This study investigates the combined effect of lauric acid and tea tree oil-loaded SLNs (LT-SLNs) with PBM at 830 nm on fibroblast cell regeneration in an in vitro diabetic wound model. Diabetic WS1 fibroblast cells were subjected to a standardised in vitro scratch assay, followed by a comprehensive evaluation of cellular responses, which included morphological alterations, wound closure dynamics, ATP production, and apoptosis at 24- and 48-hours post-irradiation. These assessments provided a critical insight into the therapeutic efficacy and cellular behaviour under experimental conditions relevant to diabetic wound healing. The results demonstrated that LT-SLNs combined with PBM exhibited no cytotoxicity at a concentration of 12.5 mg/mL. While untreated cells displayed limited wound healing capacity, treatment with LT-SLNs, particularly when combined with photobiomodulation, significantly enhanced wound closure and promoted fibroblast migration, indicating a synergistic therapeutic effect. Our study significantly enhanced wound closure, ATP production, and migratory activity, indicating a synergistic effect of both LT-SLNs and PBM. The study highlights the promising potential of LT-SLNs combined with PBM for diabetic wound healing.

**Graphical abstract:**

Exploring the combined effect of Lauric acid and Tea tree oil loaded Solid Lipid Nanoparticles (LT-SLNs) and Photobiomodulation (PBM) at 830 nm in promoting the healing of diabetic wounds through an *in vitro* fibroblast mono-layered approach. Created in BioRender. Dhilip Kumar, S. (2025) https://BioRender.com/s52u552.

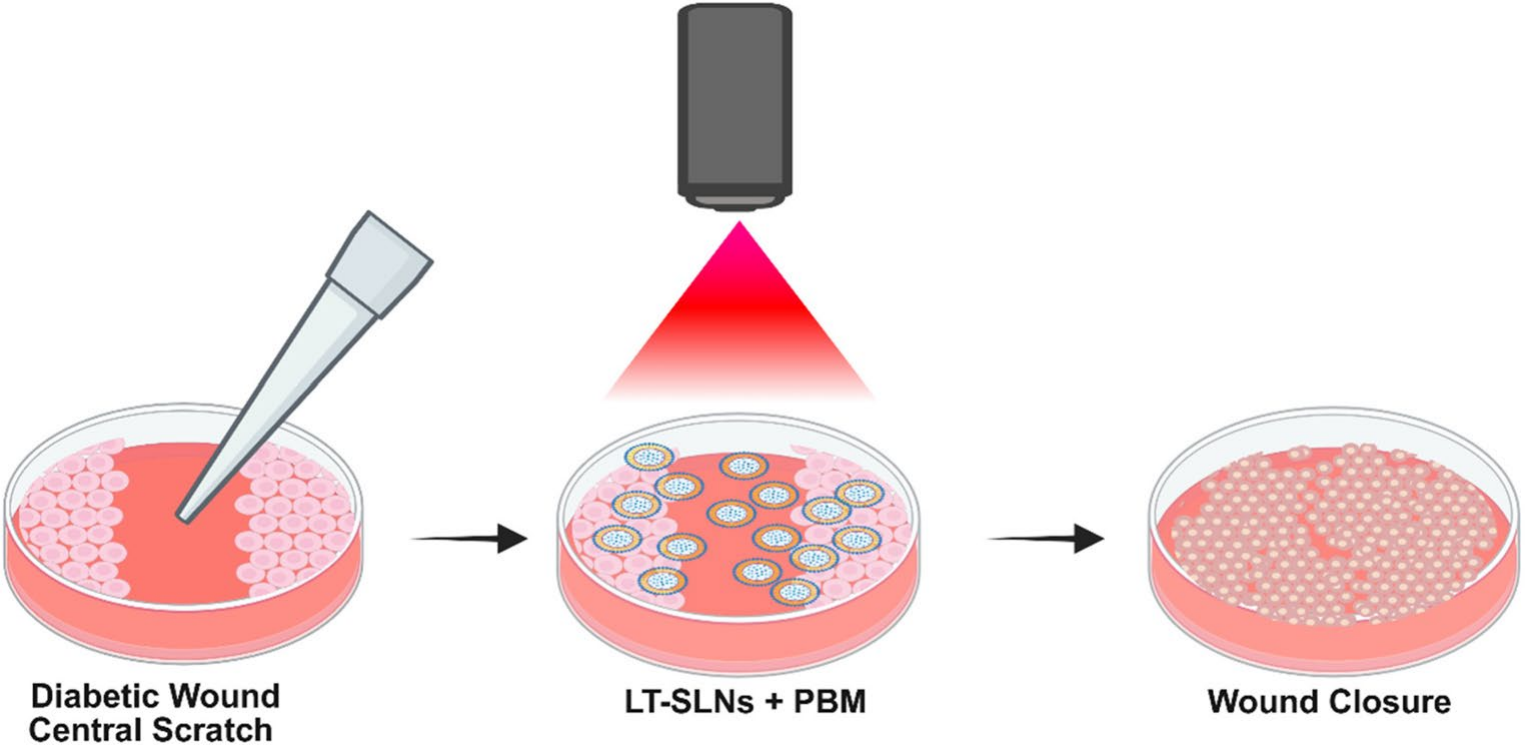

**Supplementary Information:**

The online version contains supplementary material available at 10.1007/s10103-025-04673-8.

## Introduction

Maintaining glucose levels within a narrow range is essential for glucose homeostasis, regulated by insulin and glucagon in the pancreas [1]. In the islets of Langerhans, alpha and beta cells adjust hormone secretions based on glucose levels. An imbalance can lead to hyperglycaemia, reducing insulin sensitivity and contributing to diabetes development [2]. Uncontrolled diabetes progression is linked to serious complications, including cardiovascular disease, diabetic foot ulcers (DFUs), and amputations. Over 40% of diabetic patients experience recurrent infected DFUs, which lead to two-thirds of non-traumatic amputations and have a lower 5-year survival rate than colon and breast cancer [3, 4]. Current treatments involve debridement, infection prevention, blood glucose management, and wound dressing, but adherence is often poor. This is particularly dangerous for patients with neuropathic injuries who may not feel pain, leading to worse outcomes [5]. Thus, there is a need for modern treatment techniques that improve effectiveness, reduce dosing frequency, and enhance patient compliance.

Photobiomodulation (PBM) involves the use of non-ionising red and near-infrared radiation to repair damaged tissue, alleviating pain, inflammation, and oxidative stress without thermal damage [6]. It modifies biological activity through photochemical and photophysical processes and is effective both alone and alongside other therapies [7]. PBM is beneficial for managing various chronic wounds, including those related to obstructive pulmonary disease, neuropathy, rheumatoid arthritis, and diabetic foot ulcers. Its efficacy depends on factors like energy density, light source, wavelength, and application duration [8, 9]. In diabetic wounds, PBM aids healing by reducing harmful factors through the activation of the mitochondrial enzyme cytochrome c oxidase, which stimulates the production of calcium ions, nitric oxide, and ATP, thereby decreasing reactive oxygen species and mitigating inflammation. Additionally, PBM enhances platelet activation, reduces oxidative stress, and promotes cell migration and proliferation during re-epithelialisation [10, 11].

Nanomedicines, a revolutionary class of nanoscale pharmaceuticals, face challenges like toxicity and poor drug loading, but integrating drug delivery techniques with nanostructured carriers enhances therapeutic diffusion, target-specific activation, and drug stability [12, 13]. Among these carriers, solid lipid nanoparticles (SLNs) are widely used for delivering drugs via various routes [14]. SLNs have shown significant promise in diabetic wound healing, offering improved patient outcomes. These first-generation lipid-based carriers combine the benefits of liposomes, nanoemulsions, and polymeric nanoparticles [15, 16]. Composed of a biodegradable lipid core in an aqueous surfactant, SLNs exhibit low cytotoxicity and minimal localised adverse effects. They effectively encapsulate hydrophobic and lipophilic drugs, protect them from degradation, and enable controlled release over time [14, 17]. Additionally, SLNs enhance healing in diabetic foot ulcers (DFUs) by preventing water loss, maintaining hydration, and accelerating wound closure due to their occlusive properties. Their high stability and drug-loading capacity further reduce dosing frequency while improving therapeutic outcomes [18].

Lauric acid (LA), a saturated 12-carbon fatty acid found in coconut oil, exhibits antibacterial and anti-inflammatory properties that maintain wound bed sterility and promote healing [19] However, its limitations include high molecular weight and poor water solubility, which interfere with nutrient supply and may lead to cytotoxicity at elevated concentrations, thereby impeding bioactivity at the site of injury [20]. Similarly, tea tree oil (TTO) exhibits a broad spectrum of antimicrobial properties and effectively mitigates inflammation and cellular growth [21]. Its application is limited by allergic reactions, cytotoxicity and skin irritation if not adequately formulated or diluted [22]. Therefore, this study aims to mitigate the limitations associated with the direct application of LA and TTO in the context of wound healing. This study will load LA and TTO using SLNs, enhance their stability and therapeutic efficacy and evaluate the synergistic effect of LT-SLNs combined with Photobiomodulation at 830 nm to enhance diabetic wound healing in an in vitro model, essential for understanding cellular responses during healing. In addition, the study will establish the baseline therapeutic efficacy and cytocompatibility of LT-SLNs in combination with PBM, and therefore, compare the treated groups to untreated diabetic fibroblast controls rather than to established pharmacological agents. Moreover, the 12.5 mg/ml dosage, previously identified as the minimum inhibitory concentration against *Pseudomonas aeruginosa* by Motsoene et al., [23] will be evaluated in this context to validate its dual function as both an antimicrobial threshold and a therapeutically relevant concentration for wound healing. Therefore, evaluating this dose-response framework together with the application of PBM will allow the study to validate its consistency as an optimal concentration, effectively balancing the antibacterial efficacy and high cell viability. Furthermore, the study will demonstrate the essential role of dosage in promoting essential cellular processes, including ATP production, proliferation, and wound closure.

## Methods

### Reagents

Consumables for LT-SLNs synthesis, including ethanol (99.99%), LA (98%), stearic acid (SA-98.5%), Tween 80, and TTO, were purchased from Sigma Aldrich, Johannesburg. For the diabetic in vitro cell study, additional supplies, including microscope slides, paraformaldehyde, penicillin/streptomycin, Phosphate-Buffered Saline (PBS), L-glutamine, amphotericin-β, Hank’s Balanced Salt Solution (HBSS), and Minimum Essential Medium (MEM), were also from Sigma Aldrich. TrypLE Select Enzyme and Trypan Blue stain were obtained from Invitrogen, while Rhodamine Phalloidin and BD Annexin V: FITC Apoptosis Detection Kit were supplied by The Scientific Group and Ascendis Medical, Johannesburg.

### Synthesis of LA and TTO-loaded solid nanoparticles

The SLNs and LT-SLNs formulation and optimisation were prepared based on the methodology of Motsoene et al., [23].

### Experimental models

Human skin fibroblast cells (WS1, ATCC CRL-1502™) were obtained from the University of Johannesburg’s Laser Research Centre under Research Ethics Committee approval (REC-1695-2022). Monolayers were cultured in complete Minimum Essential Media (MEM) following Ayuk et al. [24]. Diabetic WS1 cells (D-WS1) were generated by adding 17 mM/L D-glucose to the standard 5.6 mM MEM, achieving 22.6 mM. D-WS1 cells were seeded at 6 × 10⁶ cells per 35 mm culture plate and allowed to attach overnight. Diabetic wound (DW) models were created using sterile pipette tips, as described by Wurz et al. [25], with a 30-minute acclimation period before treatments. DW cells were divided into 12 groups namely: Group 1: diabetic wounded untreated (DW 0 J), Group 2: Cells + PBM (DW 5 J), Group 3: Cells + F1 (DW 0 J F1), Group 4: Cells + F1 + PBM (DW 5 J F1), Group 5: Cells + F2 (DW 0 J F2), Group 6: Cells + F2 + PBM (DW 5 J F2), Group 7: Cells + F3 (DW 0 J F3), Group 8: Cells + F3 + PBM (DW 5 J F3), Group 9: Cells + F4 (DW 0 J F4), Group 10: Cells + F4+ PBM (DW 5 J F4), Group 11: Cells + F5 (DW 0 J F5), and Group 12: Cells + F5 + PBM (DW 5 J F5).

### Morphology and percentage of wound closure

The CKX 41 microscope (Olympus, South Africa) with a digital camera (Olympus C5060-ADUS) was used to capture cell morphology. Images were taken at 4X magnification and analysed with cellSens Entry Imaging Software to assess morphology and measure wound closure (µM). Consistent image spots were used at 0, 24 and 48 h. The wound closure percentage was calculated following Kumar et al. [26] using the following equation:

$$\:Wound\:closure\:\left(\%\right)=\frac{{Pre-migration}_{length\:}-\:{Migration}_{length}}{{Pre-migration}_{length\:}}\:\times\:100$$

Where $\:{Pre-migration}_{length\:}$ is the initial distance between the edges of the central scratch at 0 h, and while $\:{Migration}_{length\:}$ illustrates the migration distance at a particular time.

### Laser irradiation

The DW cells were treated with SLN, LT-SLNs, PBM or a combination of SLNs and LT-SLNs with PBM as outlined in Table 1. PBM groups received continuous near-infrared light at 830 nm from 7 cm, with a fluence of 5 J/cm² and a power density of 11.455 mW/cm². Cellular responses were evaluated after 24 and 48 h of incubation as previously mentioned by Kumar et al. [26], 

**Table 1:** Laser parameters

Light source	Diode laser
Wavelength (nm)	830 nm
Emission	Continuous wave
Power output (mW/cm^2^)	188
Power density (mW/cm^2^)	11.455
Spot size (cm^2^)	9.1
Fluence/energy density (J/cm^2^)	5
Exposure (Time)	4 min 15 s

### ATP assay

With the aim of assessing the metabolically active (viable) cells by measuring the number of viable cells, the CellTiter-Glo^®^ 3D Cell Viability Assay (G9682, Promega, Anatech, Gauteng, South Africa) was used to detect the luminescent signal produced when ATP was converted to adenosine monophosphate (AMP) by the luciferase enzyme. In accordance with the experimental protocol established by Kumar et al., [26], cells were detached using 500 µl of pre-warmed TrypLE™ and incubated the culture for 5 min. Next, we introduced 50 µl of the ATP reagent into specific wells of a 96-well plate. The first three wells contained 50 µl of complete media without cells and served as background controls for the experiment. In addition, 50 µl of the cell suspension was dispensed into designated wells, and the plates were shielded with foil and then subjected to orbital shaking for a duration of 10 min using the polymax 1040 orbital shaker from Heidolph Instruments, Schwabach, Germany. Moreover, the Relative Light Units (RLU) luminescence levels were quantified employing a multi-plate reader (Victor3, 1420 Multilabel counter, Perkin-Elmer, Gauteng, South Africa). Subsequently, the mean background readings from the control samples were subtracted from all values documented within the treatment groups [24].

### Cell apoptosis using Annexin V-FITC/PI assay

Apoptosis was assessed using the Annexin V-FITC and PI Apoptosis Detection Kit. Annexin V-FITC identified phosphatidylserine exposure on apoptotic cell membranes, while PI staining detected membrane damage, particularly in early apoptotic cells. In accordance with the manufacturer’s guidelines, treated cell culture plates were washed thrice using HBSS, detached using 500 µl of pre-warmed TrypLE and incubated for 10 min. Subsequently, cell suspensions were resuspended in 1mL 1X binding buffer at a concentration of cells/mL. and 100 µL of the cell suspensions were aliquoted into flow cytometry tubes and combined with 5 µL each of Annexin V-FITC and PI reagents. Following the 10-minute incubation period in a light-protected environment at room temperature, the flow cytometric analysis conducted within an hour, operating at a speed of 400 events per second and a threshold set at 350 µL. The resulting quantitative data was analysed accordingly using IBM SPSS software version 27.

### Nuclear morphology and filamentous actin analysis using Rhodamine phalloidin (RP) and 4′,6-diamidino-2-phenylindole (DAPI) staining

Rhodamine Phalloidin (RP) and DAPI staining were used to assess filamentous actin and nuclear morphology. Phalloidin binds specifically to polymerised F-actin and fluoresces at 535–585 nm. DAPI stains DNA and nuclei by binding to the minor groove of DNA, activated by UV light (460–490 nm). After treating the cells for 24 and 48 h, cells were incubated with 100 µL RP for 30 min at 37 °C in a humid, dark environment, then washed with phosphate-buffered saline (PBS) and counterstained with 1 µg/mL DAPI for 10 min. Finally, the cells were fixed on a slide and analysed using a fluorescence microscope (Olympus BX41) for F-actin and nuclear morphology.

### Statistical analysis

For this research, we utilised IBM SPSS software version 27 to analyse data presented in graphs and tables. Mean values, standard errors (± SE), and P-values were employed for quantitative and qualitative results, which were conducted three times (*n* = 3). To determine statistical significance between control and treated groups, the Student t-test and One-way Analysis of Variance were employed. Additionally, we used a Dunnett test with a 95% confidence to determine statistical significance, using the p-values (*p* < 0.05*, *p* < 0.01**, and *p* < 0.001***).

## Results and discussion

### Data generation and result analysis

#### Physicochemical properties and antibacterial effect of LTSLNs

The physicochemical properties and antibacterial properties of LT-SLNs were studied and published in our recently published article [23].

#### Cellular morphology and wound closure % of W-WS1

**Fig. 1:**
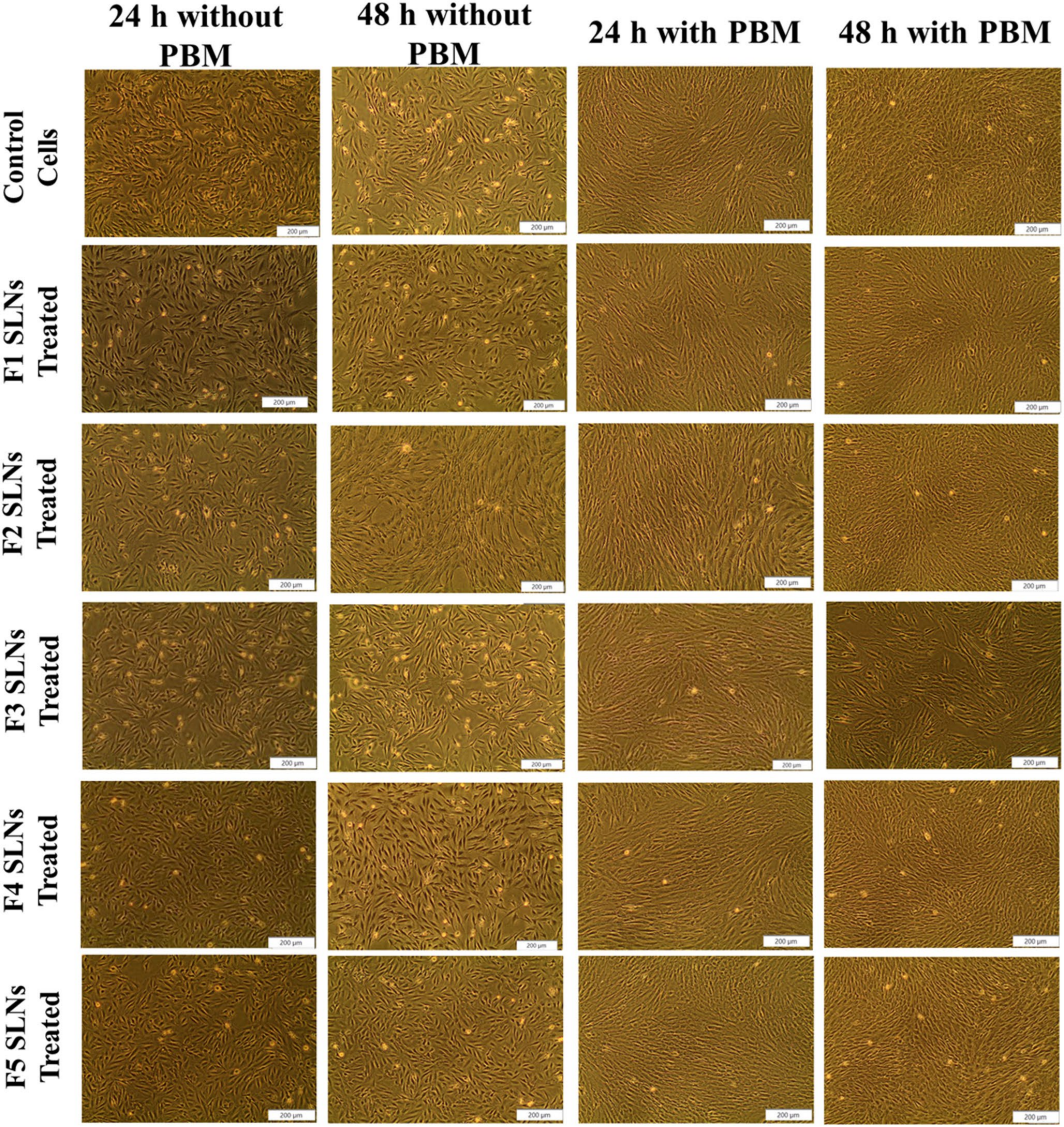
Visual representation of diabetic cell (D WS1) morphology after 12.5 mg/mL LT-SLNs treatment and LT-SLNs with PBM irradiation at 830 nm (5 J/cm²) with 24 h and 48 h incubation before analysis. All images include a scale bar representing 200 μm

The wound healing effects of LT-SLNs and LT-SLNs with PBM on diabetic cell models were examined using an inverted light microscope with a 4X magnification at 0, 24, and 48-h post-treatment to determine the cellular morphology and wound closure. The D-WS1 cells (Fig. 1.) presented a dense, homogenous monolayer with a smooth, spindle-shaped surface, typical of healthy fibroblasts. No morphological changes were observed in the treated models at 24 and 48 h compared to control groups.

**Fig. 2:**
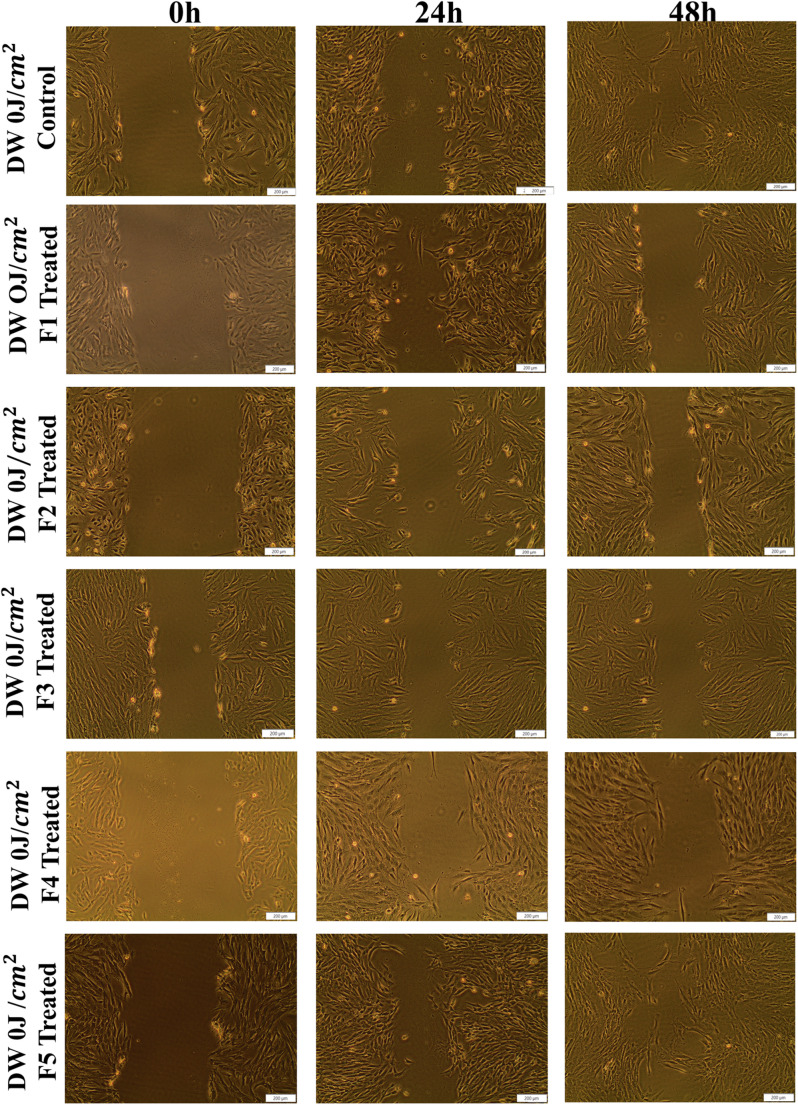
The schematic illustration displaying the DW WS1 wound closure at 0, 24, and 48-h following treatment with LT-SLNs without irradiation All images include a scale bar representing 200 μm

**Fig. 3:**
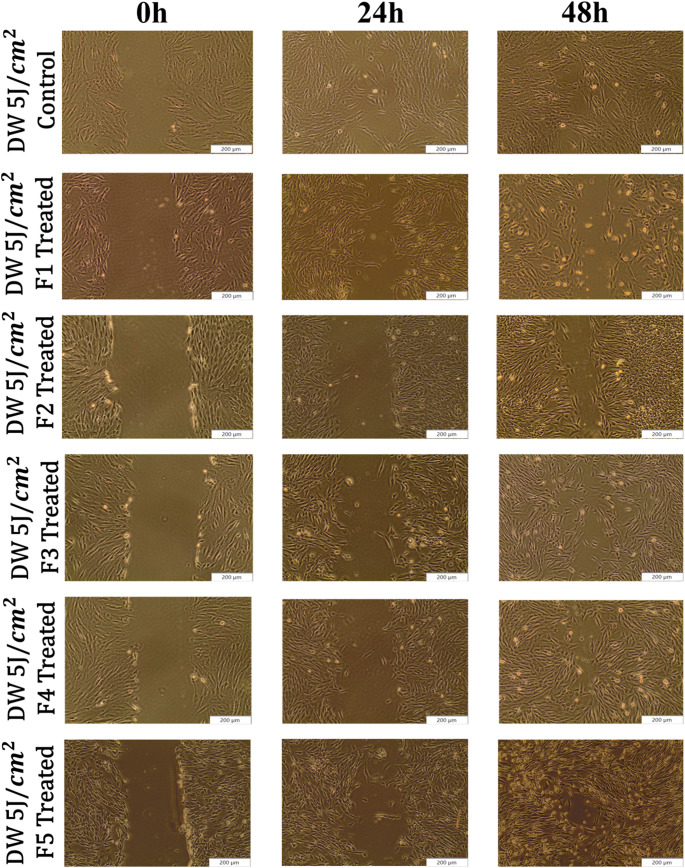
Visual representation of the wound closure in DW WS1 following treatment using LT-SLNs with PBM irradiation at 830 nm with a fluence of (5 J/cm^2^). All images include a scale bar representing 200 μm

**Fig. 4:**
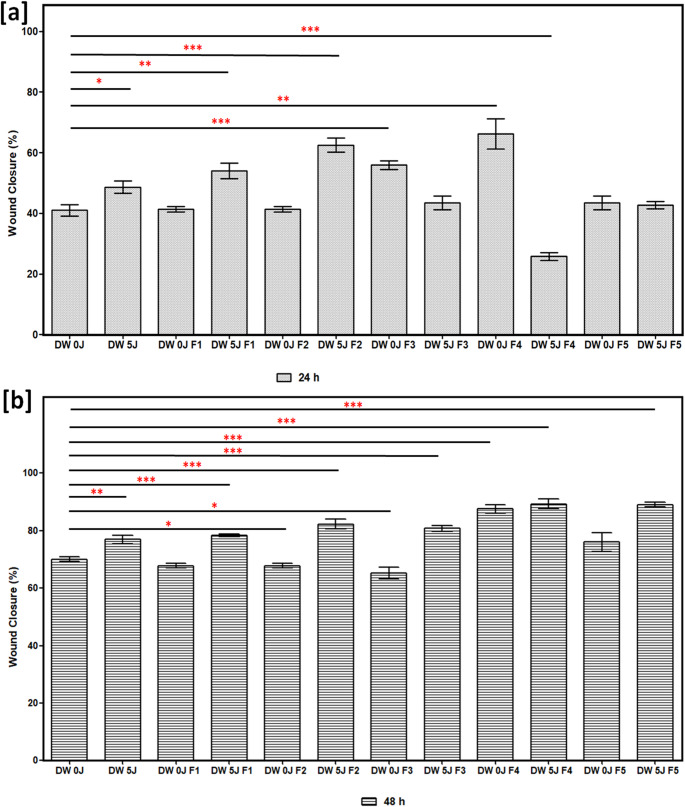
The schematic illustration displaying the DW WS1 wound closure percentage for all the formulations with irradiation (5 J/cm^2^) and without irradiation (0 J/cm^2^) at (**a**) 24 h and (**b**) 48 h, p-values (*p* < 0.05*, *p* < 0.01**, and *p* < 0.001***)

This study examined the wound closure percentages at 0, 24, and 48-h following treatment with LT-SLNs and LT-SLNs with PBM at 830 nm, using the wounded cell models shown in Figs. 2, 3 and 4. DW WS1 cells demonstrated migration toward the scratch after 24 and 48 h with significant healing observed at 48 h, particularly in LT-SLNs and PBM-treated A higher closure rate at 24 h was noted for DW 0 J F3 (*p* < 0.001), DW 0 J F4 (*p* < 0.01), DW 5 J (*p* < 0.05), DW 5 J F1 (*p* < 0.01), and DW 5 J F2 (*p* < 0.001) compared to control. No significant differences were found between control cells and other groups (i.e., DW 0 J F1, *p* = 0.607; DW 0 J F2, *p* = 0.813; DW 0 J F5, *p* = 0.312; DW 5 J F3, *p* = 0.312; and DW F5, *p* = 0.346). Moreover, 48 h, DW 5 J-treated groups (i.e. DW 0 J F4 (*p* < 0.001), DW 5 J (*p* < 0.01), DW 5 J F1 (*p* < 0.001), DW 5 J F2 (*p* < 0.001), DW 5 J F3 (*p* < 0.001), DW 5 J F4 (*p* < 0.001), and DW 5 J F5 (*p* < 0.001) demonstrated significantly higher closure percentage compared to DW 0 J cells. DW 0 J cells also had improved closure compared to DW 0 J F2 and F3 (*p* < 0.05) but no differences with other groups (e.g., DW 0 J F1, *p* = 0.480 and DW 0 J F5, *p* = 0.064).

#### ATP assay

**Fig. 5:**
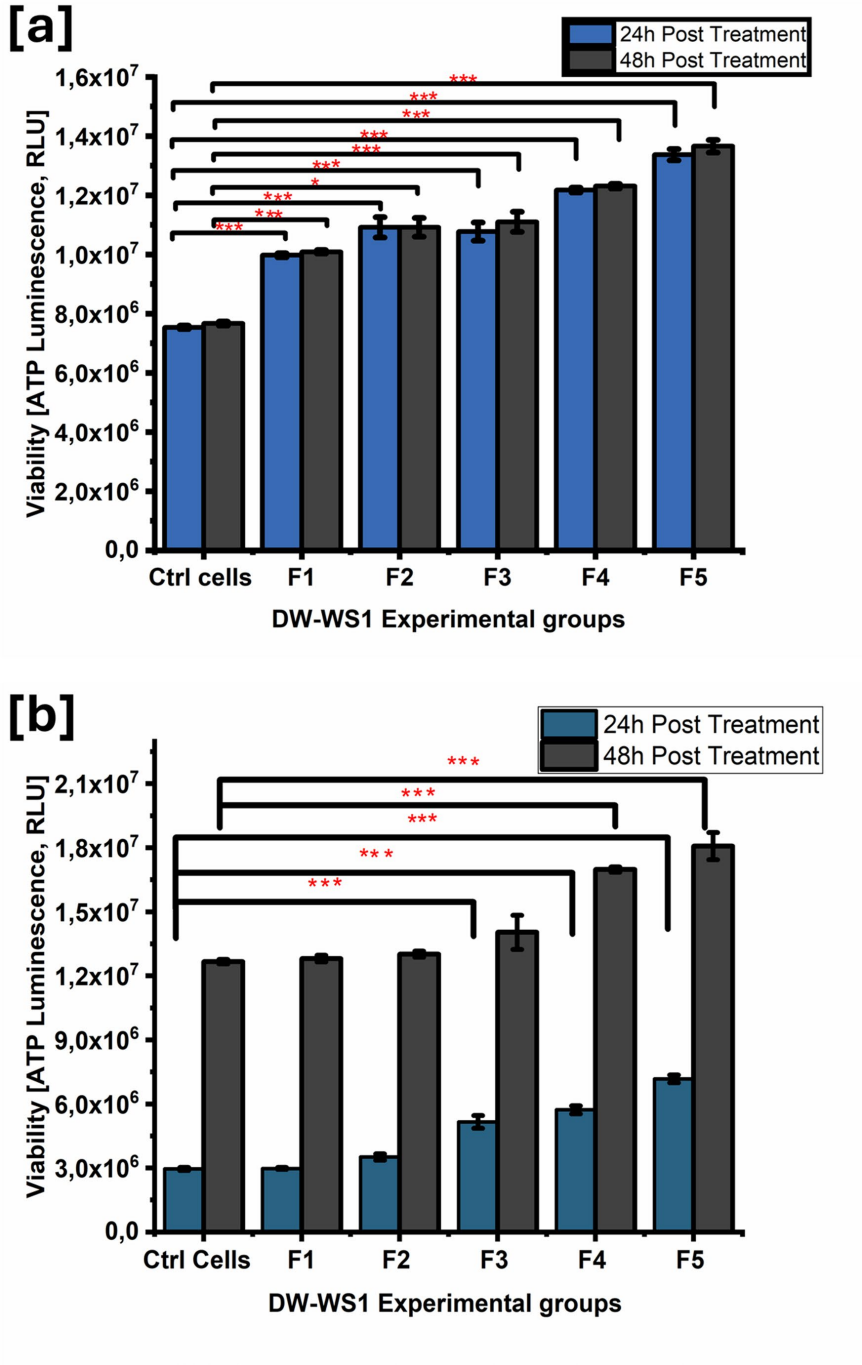
ATP levels in DW WS1 Fibroblastic cells after the [**a**] LT-SLNs and [**b**] LT-SLN with PBM at 830 nm. The values shown represent the mean ± standard errors. ***p < 0.001 indicates notable distinctions between the mean values of the control and experimental groups

The ATP CellTiter-Glo^®^ luminescent assay assessed viable cell counts after treatment with LT-SLNs and LT-SLNs combined with PBM at 830 nm. Results (Fig. 5a and b) showed a significant increase in viable cells in the diabetic wounded site at 24 and 48 h post-treatment, suggesting enhanced cellular proliferation and wound healing. Fig. 5a revealed a notable rise in ATP production in all LT-SLNs-treated models (*p* < 0.001) compared to the control over 24 and 48 h, with F5 showing the highest ATP levels. Similarly, Fig. 5b highlighted a significant increase in DW WS1 viability for LT-SLNs F3–F5 at 24 h and F4–F5 at 48 h post-treatment with PBM. While ATP levels increased in LT-SLNs F1 (*p* < 0.001), F2 (*p* = 0.165 at 24 h, *p* = 0.968 at 48 h), and F3 (*p* = 0.152 at 48 h), these changes were not statistically significant compared to the control (*p* > 0.05). LT-SLNs combined with PBM at 830 nm (0 J/cm² fluence) demonstrated the highest ATP production, indicating superior wound-healing effects over 24 and 48 h.

#### Analysis of cell death using flow cytometry (Annexin V-FITC/PI)

**Fig. 6:**
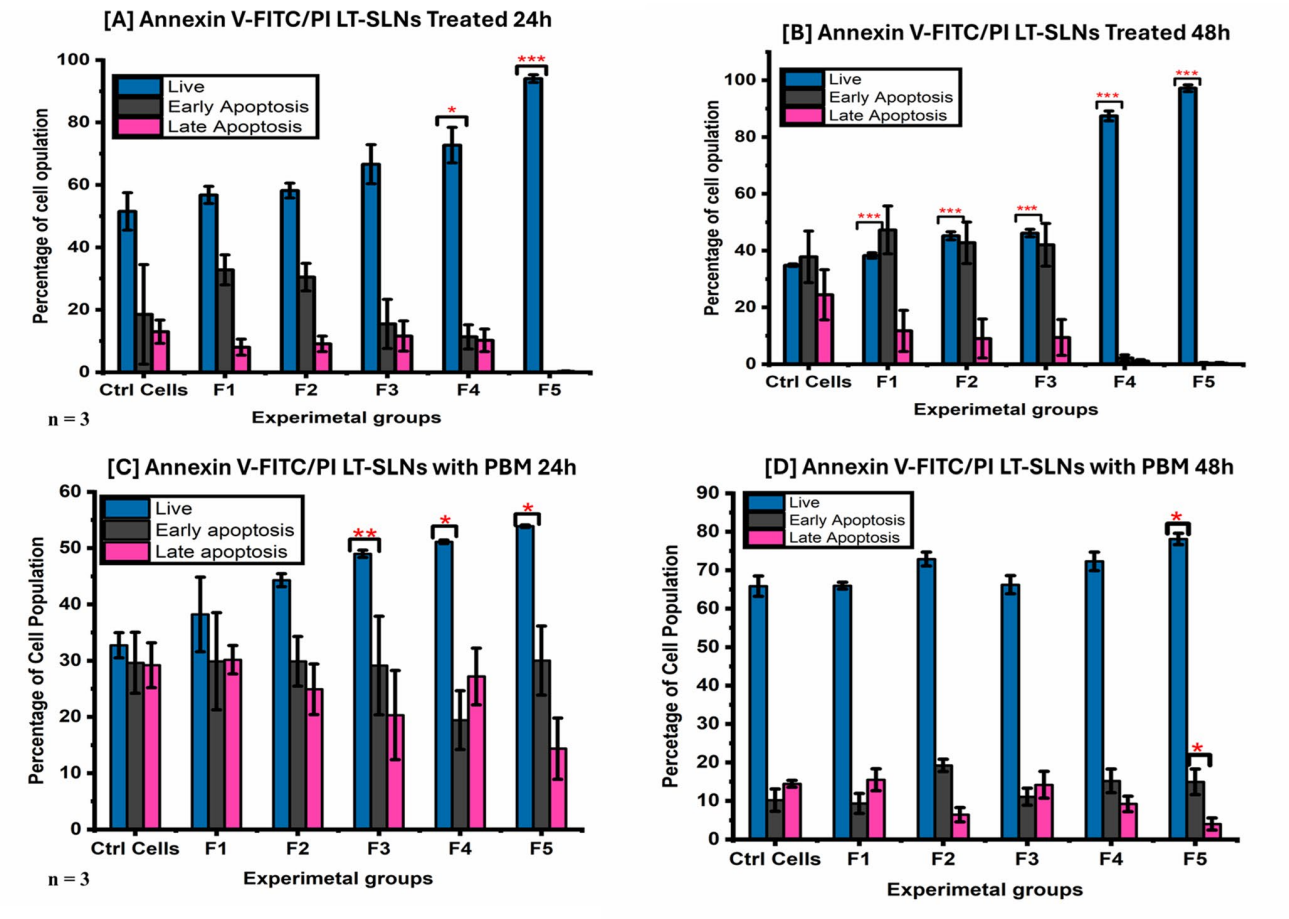
Average apoptosis rate of DW Human skin fibroblastic WS1 treated LT-SLNs without irradiation at [**A**] 24 and [**B**] 48 h and with irradiation (PBM) at [**C**] 24 and [**D**] 48 h period. The study findings provide insights into the efficacy of LT-SLNs in preventing apoptosis during diabetic wound healing with p-values (*p* < 0.05*, *p* < 0.01**, and *p* < 0.001***)

Annexin V-FITC/PI cell apoptosis detection kits were used to quantify viable cells, early and late apoptotic cells, and necrotic cells in experimental samples. The study assessed the effects of LT-SLNs alone and in combination with PBM at 830 nm on reducing early apoptosis and increasing cell viability in the DW WS1 population during wound healing. Graphical illustrations in Fig. 6 illustrate the cell population variations at 24 and 48 h. At 24 h post-treatment, LT-SLNs F4 (*p* = 0.024) and F5 (*p* < 0.001) significantly increased viable cells without notable changes in early apoptosis (EA), late apoptosis (LA), or necrosis (Fig. 6A and B). At 48 h, LT-SLNs F2–F5 significantly increased viability (*p* < 0.001), with F4 (*p* = 0.010) and F5 (*p* = 0.007) reducing early apoptosis. Scattergrams in Supplementary images 1a, 1b, 1c, and 1d supported these findings. For LT-SLNs combined with PBM at 830 nm, F3 (*p* = 0.008), F4 (*p* = 0.03), and F5 (*p* = 0.01) significantly increased viable cells at 24 h (Fig. 6C and D) with no significant changes in EA, LA, or necrosis. At 48 h, F5 increased cell viability (*p* = 0.04) while reducing late apoptosis (*p* = 0.027).

#### DAPI filamentous actin (F-Actin) and nuclear morphology analysis

**Fig. 7:**
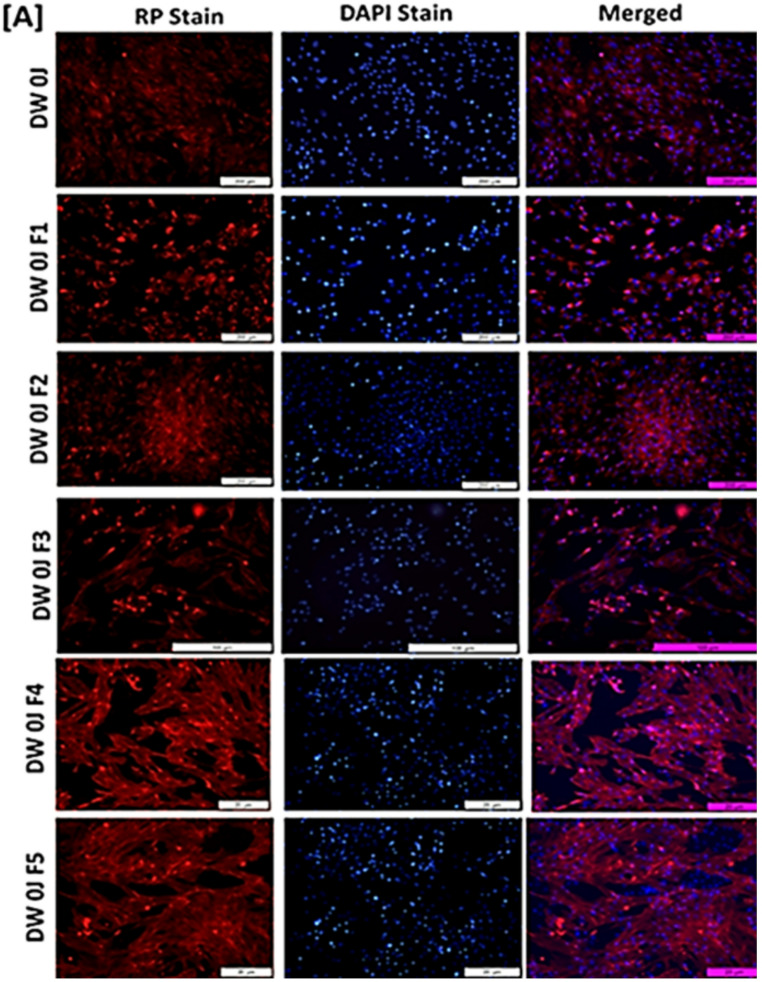

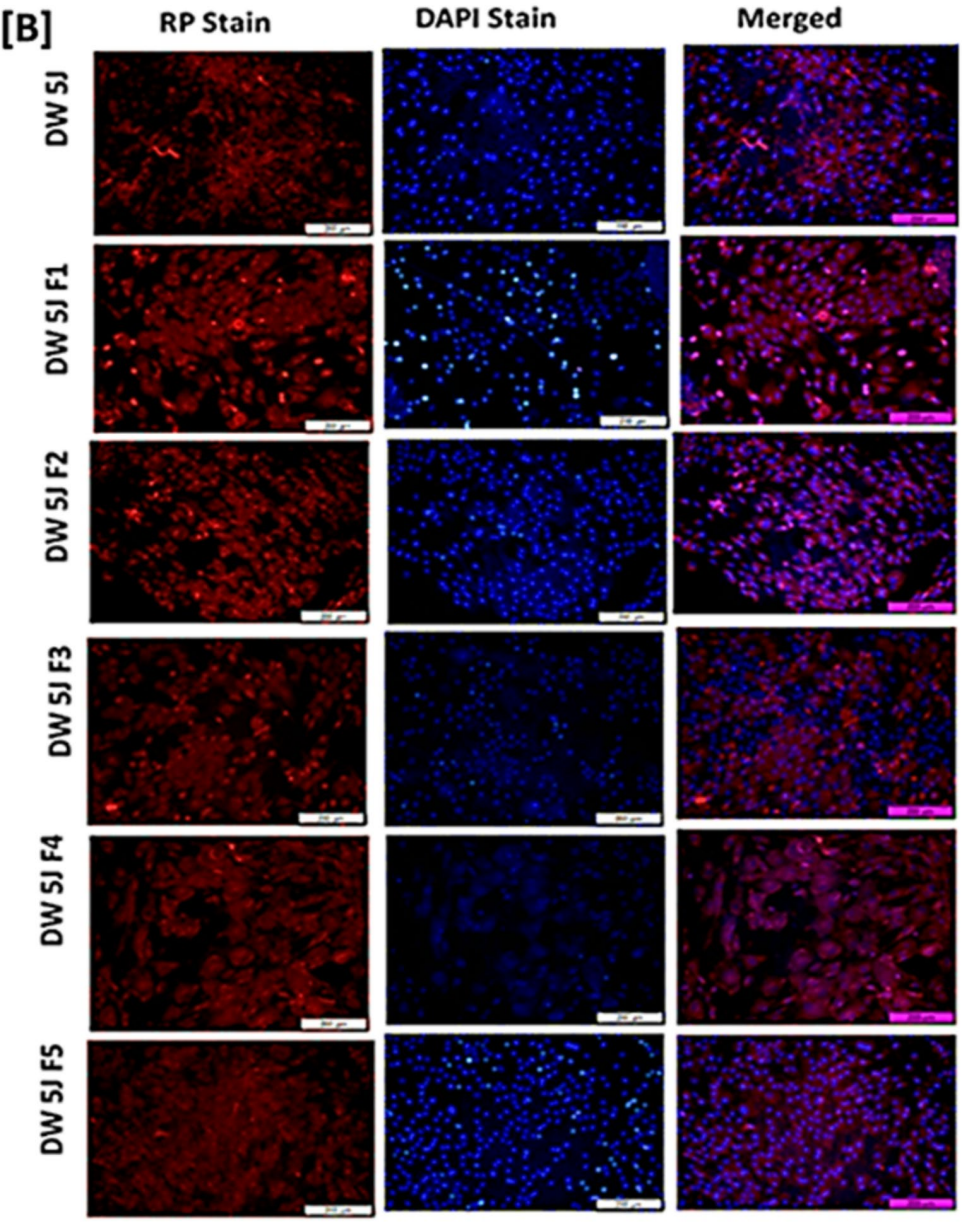
Filamentous actin (Rhodamine Phalloidin, red) and nuclear morphology (DAPI stain, blue) analysis at 24 h. The schematic illustration displays (**A**) DW Human skin fibroblastic WS1 treated with all the formulations without irradiation at 24 h and (**B**) DW Human skin fibroblastic WS1 treated with all the formulations and irradiated at 830 nm with the fluence of (5 J/cm^2^) at 24 h. All images include a scale bar representing 100 μm

In our investigation, we employed fluorescent DAPI (4′,6-diamidino-2-phenylindole) and Rhodamine Phalloidin Filamentous Actin stains, denoted in blue and red respectively, to analyse nuclear morphology and fibroblastic cell structure. The outcomes of diabetic wound healing in LT-SLNs treated non-irradiated and LT-SLNs treated with PBM at 830 nm treatment after 24- and 48-h incubation periods are visually represented in Fig. 7a and b. According to the findings, DW WS1 cells maintained their nucleus shape in both LT-SLNs treated with PBM (830 nm) and LT-SLNs treated with rhodamine-phalloidin, indicating intact nuclear integrity. Furthermore, both LT-SLNs treated with PBM and non-irradiated LT-SLNs in diabetic wounded cell models exhibited significant enhancements in F-actin fibre thickness at the 48-hour mark, likely attributable to cellular proliferation within cytoskeletal F-actin fibres during the combined therapy. Moreover, the combined effect of LT-SLNs and PBM at 830 nm wavelength with a confluence of 5 J/cm² did not manifest any adverse impacts on the diabetic wound healing process.

## Discussion

The process of tissue injury involves several stages, including coagulation and haemostasis, inflammation, proliferation, and wound remodelling, leading to scar formation. Despite advances in treatment, diabetic wounds are characterised by impaired healing stages, including reduced fibroblast function, compromised phagocytosis, and altered collagen production [26]. SLNs have emerged as a promising and practical strategy to enhance diabetic wound healing and improve patient outcomes [15]. Moreover, the application of PBM (in vitro and in vivo) wounded models has been associated with increased cell viability, proliferation, ATP growth factor, cytokine production, and nitric oxide levels, alongside reduced cell damage and pro-inflammatory cytokines resulting in the reduction of inflammation and pain while repairing tissue and nerve regeneration [9, 27]. Hence, this study aimed to overcome the challenges of using Lauric acid and Tea tree oil for wound healing by evaluating the synergetic effect of Lauric Acid and tea tree-loaded SLNs (LT-SLNs) combined with PBM at 830 nm to accelerate diabetic wound healing in vitro.

The integration of bioactive lipids and essential oils into SLNs is a promising strategy to enhance the therapeutic efficacy against drug-resistant pathogens associated with impaired wound healing, particularly in a diabetic setting. Although the therapeutic and antibacterial efficacies of LA and TTO are well documented, their direct application is often associated with degradation, instability, poor solubility and drug retention [20, 22]. Their encapsulation within SLNs not only improves their physicochemical and antibacterial effects but also facilitates controlled drug release and biocompatibility on a cellular level. In addition, the combined effect with PBM also regulates the redox stability, inflammation and cell proliferation. Consequently, the functional nanoparticles (LT-SLNs), in conjunction with PBM at a wavelength of 830 nm, present a dual therapeutic strategy that simultaneously addresses microbial burdens and enhances impaired cellular functions. With reference to the preliminary study by Motsoene et al.,, the incorporation of LA and TTO into the SLNs provided a therapeutic strategy that leveraged the drug delivery advantage of the nanocarrier and the intrinsic antibacterial effects of the bioactive compounds [23]. The moderately stable surface charge and nanoscale morphology play a crucial role in enhancing the colloidal stability, prolonged circulation, and facilitating cellular uptake, thereby ensuring therapeutic consistency [28, 29]. The observed antibacterial trends are consistent with prior research, indicating that the lipid nanoparticles can effectively disrupt bacterial membranes through direct interactions while concurrently enhancing the solubility and bioavailability of hydrophobic agents [30]. The LT-SLNs ability in inducing membrane damage in *P. aeruginosa* bacterial cells revealed a dual mode of action encompassing the destabilisation of microbial membranes by the lipid components, coupled with the enhanced effectiveness afforded by nanoscale delivery. Notably, these findings illustrated the potential of LT-SLNs not only as antimicrobial agents but also as versatile systems that can be integrated with regenerative techniques like PBM.

This study investigated the effects of LT-SLNs and LT-SLNs combined with PBM on diabetic human skin fibroblasts (D-WS1) in terms of cell migration, ATP production, proliferation, fibroblast growth, and wound closure at 0, 24, and 48 h. As such, this research article was centred on diabetic WS1 fibroblasts as they are widely recognised as a reliable in vitro model for diabetic wound healing due to their diminished proliferative and migratory abilities, increased oxidative stress, and altered extracellular matrix production [31], in addition, the study established a baseline efficacy and cytocompatibility of the LT-SLNs in combination with PBM, through the use of untreated D-WS1 cell models rather than established pharmaceutical agents silver sulfadiazine. Microscopic analysis showed consistent cell morphology across all experimental conditions, confirming low toxicity of LT-SLNs and LT-SLNs combined with PBM. Moreover, D-WS1 cells exhibited a characteristic morphology characterised by flat, slender, and spindle-shaped bodies, featuring bi- or multi-polar projections, a consistent observation with the findings of Ayuk et al. [24], and Jere et al. [31],. Furthermore, Wound closure, driven by haptotaxis and chemotaxis signals, was supported by MEM media, providing optimal nutrition for cell growth and functionality [32, 33].

SLNs enhanced drug solubility, stability, and sustained release of LA and TTO with low toxicity, while their lipid composition facilitated cell membrane diffusion, promoting growth, reducing inflammation, and accelerating wound healing [18]. The cell migration toward the central scratch (Figs. 3, 4 and 5.) increased significantly, with irradiated LT-SLNs showing faster wound closure [34]. Encapsulated Lauric Acid and Tea Tree Oil enhanced fibroblast proliferation, aligning with previous studies [35, 36]. Hyperglycaemia, a major barrier to diabetic wound healing, reduces fibroblast activity, but treatments with LT-SLNs and PBM improved cell viability, proliferation, and ATP levels, as shown by the CellTiter-Glo assay and Live/Dead apoptosis assay. Additionally, the above studies emphasised the crucial role of fibroblasts in tissue disc and regeneration, while the D-WS1 cell treatment models revealed a significant increase in the vitality of regenerated cells during ATP evaluation post-treatment. Moreover, formulations F4 and F5 showed the highest ATP levels in PBM-treated models, which was further supported by the Live/Dead cell apoptosis assay that indicated a positive rise in vitality among diabetic wound models post-treatment. As immunofluorescence is a valuable tool for studying cellular biological processes, enabling visualisation of intercellular and intracellular events as well as organelle structures. It revealed the preserved nuclear morphology and increased F-actin fibre thickness in treated cells, indicating cytoskeletal reinforcement during wound healing. Furthermore, the combined therapy with LT-SLNs and PBM maintained cellular integrity without hindering wound healing, highlighting its therapeutic potential and safety for diabetic wound management.

While the study provides a valuable insight into the combined effect of LT-SLNs and PBM in diabetic in vitro cell models, certain design considerations limit the extent of its translational applicability. This study highlights the therapeutic promise of integrating bioactive and functionalised LT-SLNs with PBM as a dual-modality approach in treating diabetic wound healing. Consequently, this study primarily focused on the functional outcomes and cellular responses to demonstrate the combined therapeutic potential of LT-SLNs with PBM at 830 nm. The results obtained consistently indicated mechanistic activation, as supported by several key findings: (i) the combined treatment significantly enhanced wound closure, demonstrated by the contraction of the central scratch in monolayer cultures within 24–48 h, (ii) this was accompanied by high cell viability, increased ATP levels, and evidence of proliferative and angiogenic responses, all of which are well-documented downstream outcomes of cytochrome c oxidase activation during PBM, and (iii) these observations are strongly aligned with prior literature showing that PBM-mediated mitochondrial activation and ROS modulation lead to ATP upregulation, triggering cell proliferation, migration, and angiogenesis. However, the study did not necessitate a comparison against well-established pharmaceutical standards. Moreover, direct protein/gene expression limited the depth of this research, together with the interpretation of metabolic mechanisms. The novelty of our study demonstrated the synergistic interaction between phytochemical-loaded SLNs and PBM in enhancing wound healing processes. Future studies will use RT-qPCR and Western blotting to confirm the regulation of key signalling pathways (e.g., VEGF, HIF-1α, and mitochondrial respiratory chain proteins) to validate these hypotheses. Furthermore, the concerns surrounding the long-term biocompatibility of LT-SLNs will be emphasised in the formulation process, as well as in their translation for in vivo and clinical applications focused on diabetic wound healing.

## Conclusions

The present study aimed to investigate the efficacy of combining LT-SLNs and PBM against D WS1 wound cells in an in vitro. The results demonstrated that the combination of LT-SLNs with PBM improved therapeutic efficacy by promoting cell proliferation and wound closure while suppressing cell death. The diabetic DW WS1 cells did not exhibit any significant nuclear and morphological changes following treatment with LT-SLNs without irradiation and with irradiation, indicating the safety of the approach. Comparatively, LT-SLNs DW WS1 cells had a better wound healing rate, resulting in partially closed wounds in LT-SLNs (F4 and F5). The ATP assay revealed a significant increase in ATP production of irradiated cells with LT-SLNs (F5, 5 J/cm^2^) at both 24 and 48 h. Furthermore, the formulation F5 exhibited greater tolerance for diabetic wound healing at 24 and 48 h after the combined treatment with PBM, resulting in an increased live cell count of the F5-treated groups compared to other formulations. Based on the comprehensive literature and outcomes from the present study, the synergistic effects of LT-SLNs and PBM at 830 nm at a light dose of 5 have the potential to become an alternative approach for complementary medicine in the treatment of diabetic wounds. While this model effectively highlights the therapeutic potential under hyperglycaemia-induced stress, it does not include a direct comparison with fibroblasts maintained under normoglycemic conditions. Future studies incorporating such normoglycemic conditions and the use of standard wound-healing agent (Silver sulfadiazine as a positive control) will facilitate further delineation of the specific impact of elevated glucose levels and strengthen the translational relevance of the findings. However, it is recommended to conduct more in vitro studies and in vivo animal model studies, and clinical trials to explore the full potential of LT-SLNs combined with PBM and unlock more interesting outcomes on this formulation.

## Supplementary Information

Below is the link to the electronic supplementary material.

Supplementary file 1 (DOCX 2.93MB)

## Data Availability

The authors declare that the data supporting the findings of this study are available within the paper. Should any raw data files be needed in another format they are available from the corresponding author upon reasonable request.
