# Supplementary material for: Synergetic effect of lauric acid and tea tree oil-loaded solid lipid nanoparticles and photobiomodulation in diabetic wound healing
Source: Lasers Med Sci. 2025 Oct 11;40(1):427. doi: 10.1007/s10103-025-04673-8 (PMC12515205; doi:10.1007/s10103-025-04673-8)

**Supplementary figure 1a to 1d**

**Figure 1A.**. Annexin V-FITC/PI scattergrams illustrating the cell death of diabetic Human skin fibroblastic WS1, illustrating analysis at 24h post LT-SLN treatment


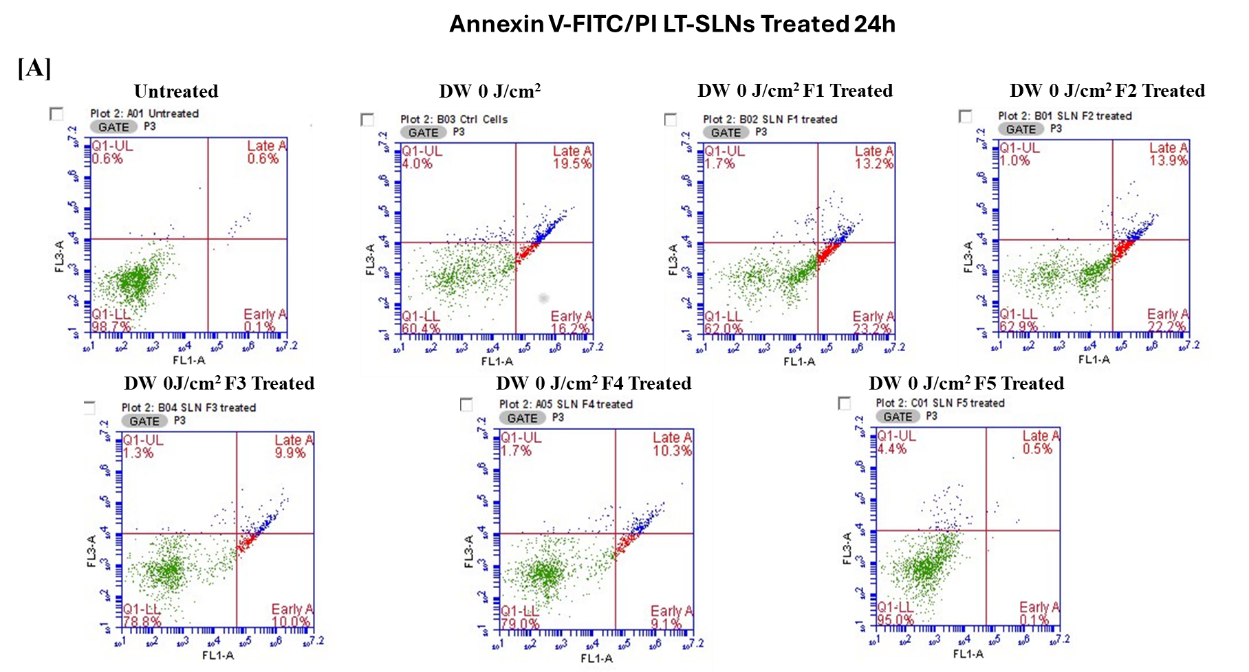


**Figure 1B**. Annexin V-FITC/PI scattergrams illustrating the cell death of diabetic Human skin fibroblastic WS1, illustrating analysis at 24h post LT-SLN treatment


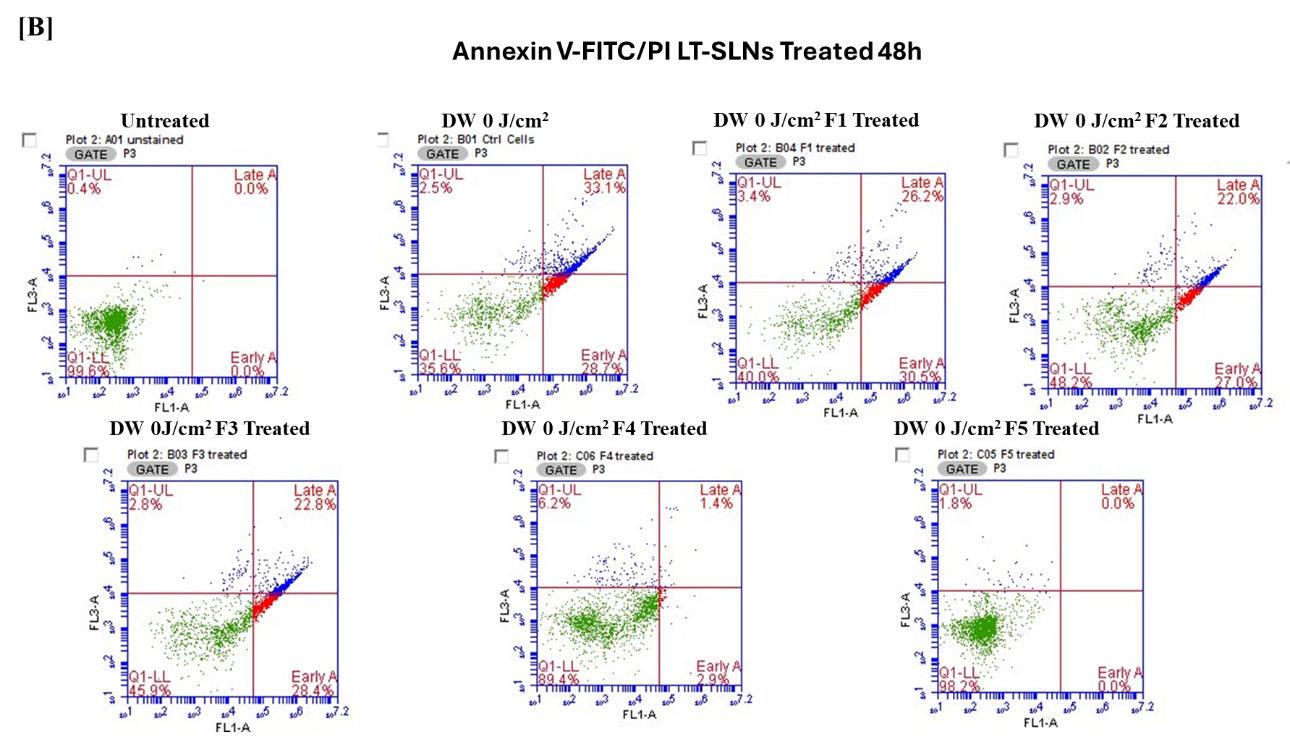


**Figure 1C**. Annexin V-FITC/PI scattergrams illustrating the cell death of diabetic Human skin fibroblastic WS1, illustrating analysis at 24h post LT-SLN treatment with PBM at 830 nm


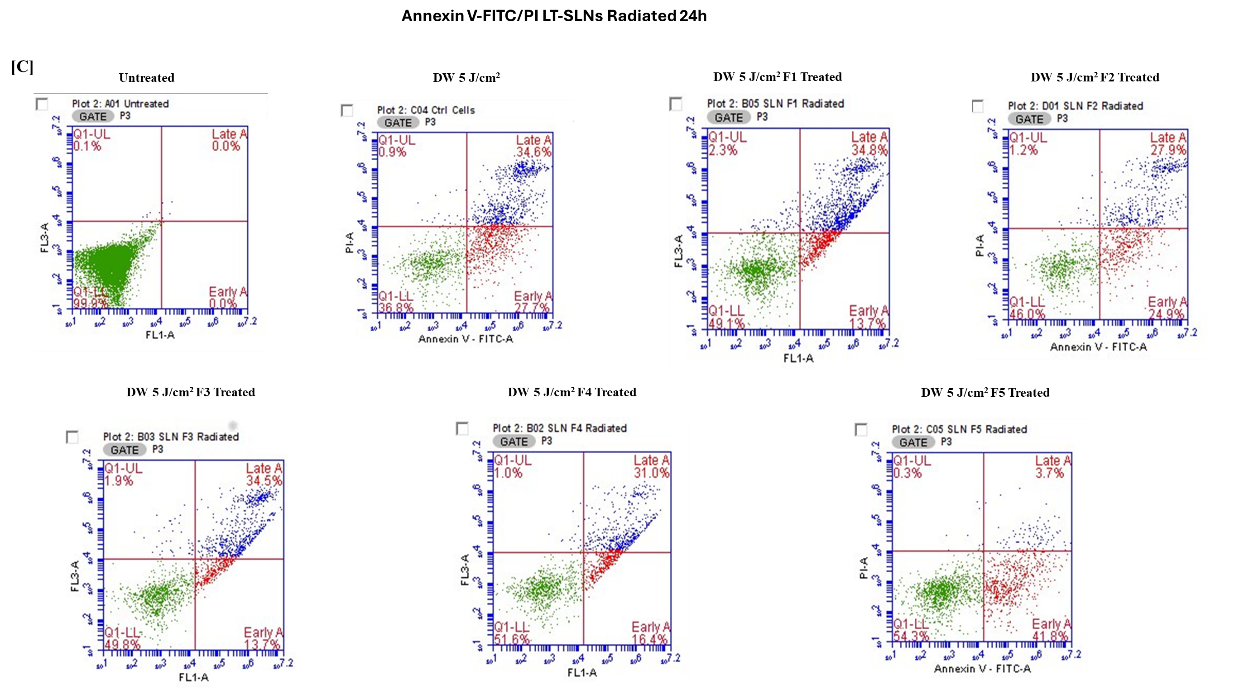


**Figure 1D.** Annexin V-FITC/PI scattergrams illustrating the cell death of diabetic Human skin fibroblastic WS1 illustrating analysis at 48 h post-LT-SLNs with PBM


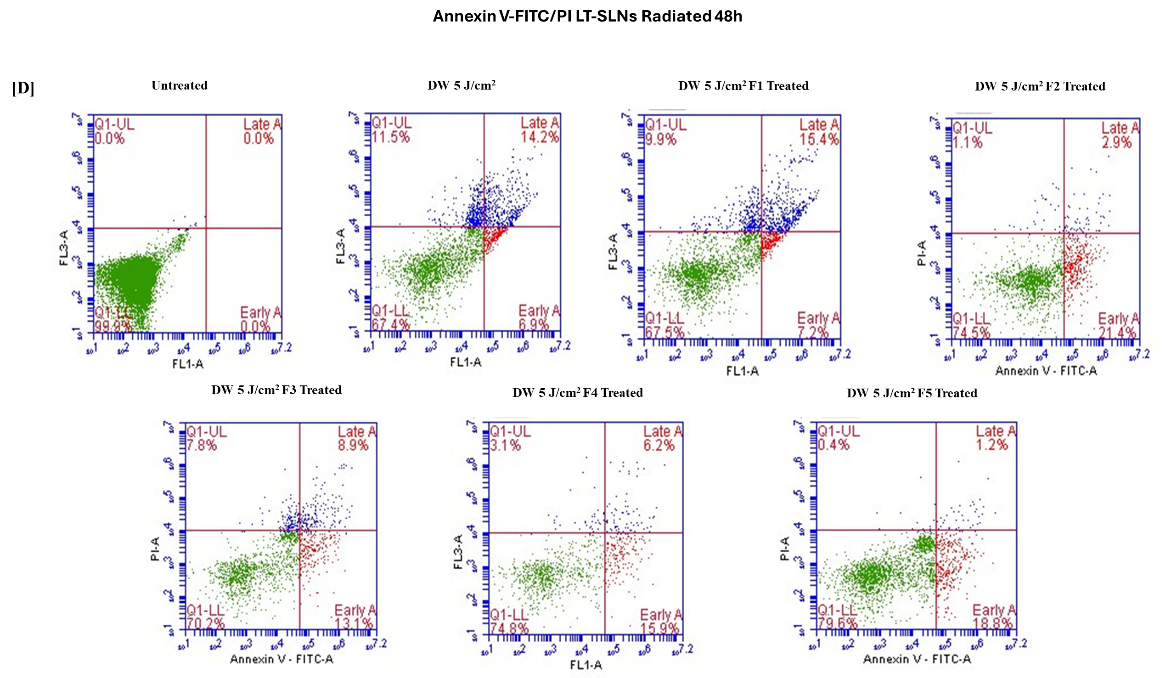

Supplement: Supplementary file 1 — (DOCX 2.93MB) [file 10103_2025_4673_MOESM1_ESM.docx]
